# Order through destruction: how ER‐associated protein degradation contributes to organelle homeostasis

**DOI:** 10.15252/embj.2021109845

**Published:** 2022-02-16

**Authors:** John C Christianson, Pedro Carvalho

**Affiliations:** ^1^ Nuffield Department of Orthopaedics, Rheumatology and Musculoskeletal Sciences Botnar Research Centre University of Oxford Oxford UK; ^2^ Sir William Dunn School of Pathology University of Oxford Oxford UK

**Keywords:** endoplasmic reticulum, ERAD, protein degradation, protein quality control, ubiquitin ligase, Organelles, Post-translational Modifications & Proteolysis, Translation & Protein Quality

## Abstract

The endoplasmic reticulum (ER) is a large, dynamic, and multifunctional organelle. ER protein homeostasis is essential for the coordination of its diverse functions and depends on ER‐associated protein degradation (ERAD). The latter process selects target proteins in the lumen and membrane of the ER, promotes their ubiquitination, and facilitates their delivery into the cytosol for degradation by the proteasome. Originally characterized for a role in the degradation of misfolded proteins and rate‐limiting enzymes of sterol biosynthesis, the many branches of ERAD now appear to control the levels of a wider range of substrates and influence more broadly the organization and functions of the ER, as well as its interactions with adjacent organelles. Here, we discuss recent mechanistic advances in our understanding of ERAD and of its consequences for the regulation of ER functions.

## Graphical abstract

Christianson and Carvalho review the mechanisms and roles of ERAD, from original implications in protein quality control and sterol biosynthesis regulation to emerging functions in shaping the ER and its interactions with other compartments.

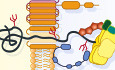

## Introduction

The specialized functions and environments of cellular organelles are highly dependent on their unique protein compositions and how these resident proteomes are maintained. While this is established by elaborate protein targeting mechanisms that guide newly synthesized molecules to the correct organellar destination, the proteomes of individual organelles are unique and dynamic, continuously being shaped and maintained by a variety of quality control systems working in parallel. These systems simultaneously promote the retention of appropriate forms and the clearance of proteins whose folding, assembly, localization, or abundance is incorrect. Thus, protein quality control systems play a central role in organellar and cellular homeostasis. Highlighting their importance, defects in quality control components are often incompatible with life or optimal viability, or result in devastating diseases (Bhattacharya & Qi, 2019; Needham *et al*, 2019).

As a large, multifunctional organelle, the endoplasmic reticulum (ER) houses robust protein quality control systems, on which it relies heavily. First and foremost, the ER is the main site for membrane protein and secreted protein biogenesis, lipid biosynthesis, and for storage of intracellular calcium. The ER also accommodates proteins and protein complexes required for many other cellular functionalities including innate immune signaling, stress responses, and metabolism. These activities take place throughout a continuous network of sheet‐like cisternae and dynamic membrane tubules, defining a single luminal compartment (Westrate *et al*, 2015). Moreover, the extensive structure of the ER impacts the overall architecture and organization within eukaryotic cells. For example, the nuclear envelope, essential for defining the nucleus and its organization, is formed by apposed ER sheets—the inner and outer nuclear membranes—separated by the ER lumen. Also, ER tubules extending into the cytoplasm contact other organelles such as mitochondria and endosomes, thereby influencing both their positioning and dynamics. Thus, spatially coordinating (and compartmentalizing) these concurrent processes requires the composition of various ER domains to be continuously surveyed by protein quality control systems.

The best‐characterized protein quality control system of the ER is the ubiquitin‐proteasome‐dependent process of ER‐associated protein degradation (ERAD). While extensively linked to the degradation of unwanted byproducts of protein biogenesis, such as misfolded and unassembled/orphaned proteins, it is now clear that ERAD and the ubiquitination machineries on which it relies also play important roles in continuously shaping the established ER proteome, and consequently its multiple functions. By promoting the regulated degradation of various ER‐localized proteins, ERAD exerts an aspect of post‐translational control over lipid homeostasis, nuclear envelope organization, inter‐organelle contact (e.g., mitochondria, endosomes), calcium flux, and even innate immune signaling. Autophagy‐dependent and ‐independent clearance through endo‐lysosomes (Fregno & Molinari, 2019; Molinari, 2021), or direct extraction of mislocalized membrane proteins (McKenna *et al*, 2020; Dederer & Lemberg, 2021), are alternative strategies for regulating ER composition. Here, we will however focus on the recent advances in understanding the fundamental mechanisms of ERAD from studies both in yeast and mammalian cells. We will also discuss recent findings that highlight the contributions of ERAD and ubiquitination machinery to the control of ER functions and association with proximal organelles.

## The fundamentals of ERAD

**Figure 1:**
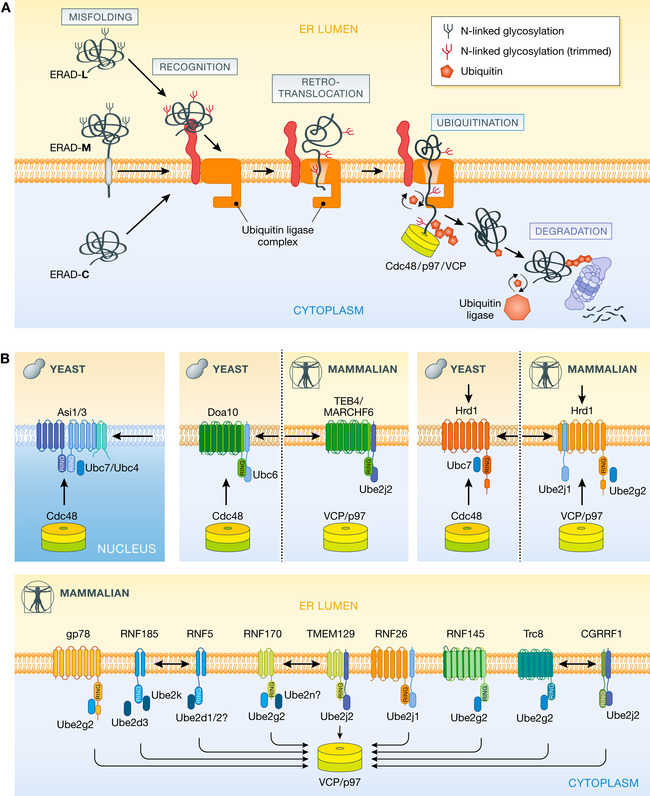
The steps and ubiquitin ligases involved in ERAD (A) General steps involved in the ERAD of different classes of misfolded proteins (in black). Ubiquitin ligase complexes in the ER membrane promote the recognition, retrotranslocation, and ubiquitination of ERAD substrates. Recognition of misfolded glycoproteins in the ER lumen requires trimming of glycans (in red, see text for details). Ubiquitinated substrates are released from the ER membrane into the cytosol by the Cdc48/p97/VCP ATPase, trimmed, and re‐ubiquitinated before being handed to the proteasome for degradation. (B) E3 ubiquitin ligases and their cognate E2 enzymes characterized in yeast and mammalian cells. Homologous ERAD complexes in yeast and mammalian cells are shown together, separated by dotted lines. ERAD complexes exclusive to yeast and mammalian cells are show in separate boxes. Arrows indicate the subcellular environment by which substrates have been shown to access each E3.

The ERAD pathway of both yeast and mammalian cells is comprised of multiple branches, each operating in parallel through a membrane‐embedded ubiquitin ligase complex. Each ERAD branch (or complex) engages substrates through recognition domains located in either the lumen or membrane of the ER, facilitating substrate retrotranslocation (or relocalization) into the cytosol where the ubiquitination machinery resides. Soluble secretory proteins confined entirely by the ER lumen must (at least partially) unfold in order to traverse the lipid bilayer for ubiquitination. Both mono‐ and polytopic membrane substrates, with domains already exposed to the cytoplasm, are afforded the opportunity to be ubiquitinated even before retrotranslocation of their membrane and luminal domains (Fig 1A). Once ubiquitinated by their respective ubiquitin ligase complex, substrates of all ERAD branches converge on a cytosolic AAA‐ATPase known as Cdc48 in yeast and p97/VCP in mammals, which aids in the process of retrotranslocation by engaging and “pulling” ubiquitinated substrates from the grasp of the ER membrane, before finally handing them off to the 26S proteasome for degradation.

While all ERAD branches are thought to abide by this generic framework, the distinct mechanisms by which most ERAD branches select, differentiate, and retrotranslocate substrates remain largely unknown (see below). The key role of 26S proteasomes as the ultimate destination in substrate destruction in ERAD has long been established (Ward *et al*, 1995; Hiller *et al*, 1996). Similarly, the essential role Cdc48/p97/VCP plays upstream to efficiently deliver substrates to the proteasome in ERAD is well documented, with its genetic disruption or pharmacological inhibition causing ERAD substrates to accumulate (Bays *et al*, 2001; Ye *et al*, 2001; Jarosch *et al*, 2002; Rabinovich *et al*, 2002; Chou *et al*, 2011). Positioned between the ubiquitin conjugation machinery at the ER membrane and cytosolic proteasomes, Cdc48/p97/VCP provides a common avenue for the continuous processing and transfer of ERAD substrates.

Cdc48/p97/VCP access to substrates emerging from the ER is enhanced by its direct recruitment to ER membranes through factors containing VCP‐interacting motifs. In yeast, an N‐terminal UBX domain of the ER membrane protein Ubx2 serves as the Cdc48 tether (Neuber *et al*, 2005; Schuberth & Buchberger, 2005). Likewise, in metazoans, UBX domains as well as other p97/VCP‐interacting motifs (e.g., SHP, VIM, VBM) can be found within ERAD complex components such as Derlin‐1/2 and UBXD8, and even within E3s themselves (e.g., Hrd1, gp78/AMFR) (Ballar *et al*, 2006; Morreale *et al*, 2009; Greenblatt *et al*, 2011). By recruiting Cdc48/p97/VCP to sites of retrotranslocation, these domains could be seen to facilitate continuous processing of ERAD substrates, expediting their removal from the ER and their delivery to proteasomes. However, not all ER‐resident E3 complexes enriched for p97/VCP appear to contain canonical VCP‐interacting motifs (Fenech *et al,* 2020). It is unclear whether yet unidentified interacting motifs are present within these ERAD branches, or whether the polyubiquitin adducts on substrates themselves might be interacting directly with p97/VCP cofactors such as Npl4 (Stein *et al*, 2014). IP‐MS studies have revealed that association of p97/VCP with ER‐resident E3s varies greatly, being highly enriched in a subset of E3s while absent or only very modest for a few other E3s (Hülsmann *et al*, 2018; Fenech *et al*, 2020). One interpretation could be that p97/VCP enrichment with ER‐resident ubiquitin ligases serves as an indicator of substrate flux through individual ERAD branches. In this scenario, ER‐resident E3s that are not enriching p97/VCP may be performing functions that are beyond the scope of protein degradation. Deciphering the variety of roles being played here by ER‐resident E3s will be an important area for future studies.

### Just passing through: ERAD substrate engagement with Cdc48/p97/VCP

Although the general role of Cdc48/p97/VCP in ERAD has been appreciated for some time, several important recent advances have helped to understand how this ATPase is able to engage diverse substrates originating from all ERAD branches (Fig 1A and B) as well as other cellular pathways.

**Figure 2:**
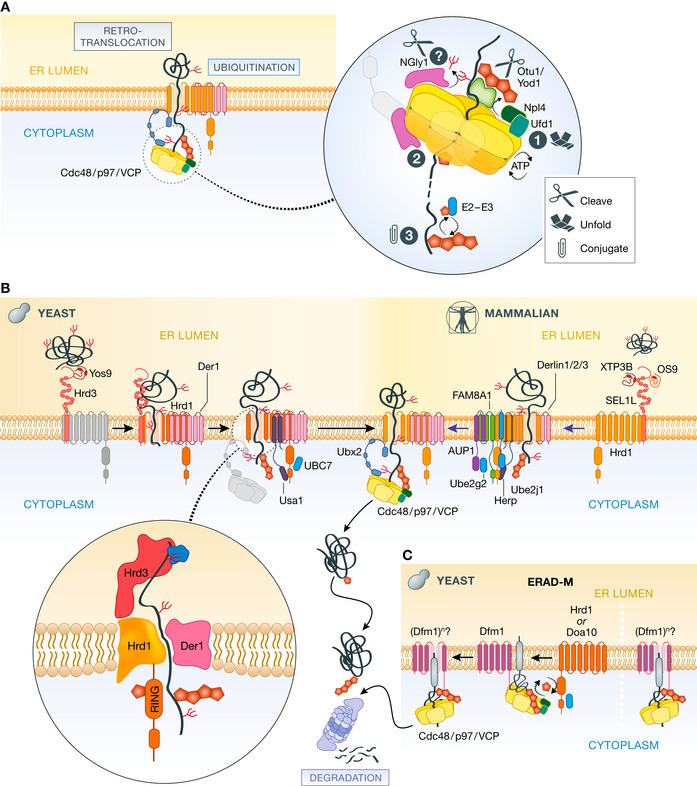
Proposed mechanisms for key processing steps in ERAD (A) Cdc48/p97/VCP ATPase (yellow) has a universal role in pulling ubiquitinated substrates from the ER membrane. Ubiquitin molecules conjugated to the substrate interact with the ATPase complex and unfold as they enter the central pore (1). Rounds of ATP hydrolysis promote the threading of the substrate through the central pore (2). As substrates emerge on the trans side of the ATPase complex, they may be re‐ubiquitinated by soluble ubiquitin ligases (3). De‐ubiquitinating enzymes (Otu1 and Yod1 in yeast and mammals, respectively) on the *cis*‐side remove residual ubiquitin moieties, facilitating the remaining residues of the substrate to travel through the central pore. Glycans conjugated to glycoproteins are removed by peptide *N*‐glycanase enzymes (Png1 and NGly1 in yeast and mammals, respectively) associated with the *cis*‐ or *trans*‐sides of the Cdc48/p97/VCP complex. (B) Proposed mechanisms for the retrotranslocation of luminal glycoproteins. In yeast (from left to right), upon recognition by Hrd3/Yos9, substrates engage with the membrane domains of Hrd1 and Der1, which form two halves of a channel. Besides being rich in hydrophilic residues, Hrd1/Der1 membrane regions promote the local thinning of the ER membrane, thereby facilitating the retrotranslocation of the substrate to the cytosolic side of the ER membrane, where it is ubiquitinated by the activity of Hrd1 RING domain (in inset). Ubiquitinated substrate is pulled through the channel and across the membrane by the Cdc48/p97/VCP as described in (A). Although less mechanistic studies are available, analogous steps are thought to occur during the retrotranslocation of luminal glycoproteins in mammals (Right to left). (C) Proposed retrotranslocation mechanism for a membrane ERAD substrate in yeast. Membrane substrates ubiquitinated by the Hrd1 or Doa10 ERAD ubiquitin ligase complexes interact with Dfm1, which facilitates their retrotranslocation with the assistance of the Cdc48/p97/VCP ATPase complex. Dfm1 can also promote retrotranslocation of ubiquitinated substrates independently of Hrd1 and Doa10. The mechanisms and the oligomeric state of Dfm1 during retrotranslocation are unclear.

Cdc48/p97/VCP is an abundant and multifunctional AAA‐ATPase. Monomers of Cdc48/p97/VCP assemble to form a hexamer with two stacked rings (D1/D2) defined by a central pore. Cdc48/p97/VCP recruits the co‐factors Npl4/Ufd1, which bind asymmetrically to one side of the ring (*cis*/D1). Npl4/Ufd1 facilitates recruitment of polyubiquitinated substrates to the complex by interacting directly with their ubiquitin chains (Sato *et al*, 2019). Interestingly, Npl4 interaction reportedly leads to the unfolding of one of the ubiquitin molecules within the chain as it inserts into a Npl4 groove (Cooney *et al*, 2019; Twomey *et al*, 2019). This unfolded ubiquitin molecule can then reach the central pore of the Cdc48/p97/VCP ring through which the substrate is threaded (Fig 2A). Translocation of substrates through the Cdc48/p97/VCP central pore is powered by the aromatic residues that line it, which change conformation upon ATP hydrolysis, as has been observed in related ATPases including the 19S proteasome (de la Peña *et al*, 2018) and bacterial Clp ATPases (Martin *et al*, 2008; Rizo *et al*, 2019). Coordination of the ATPase cycles of the six Cdc48/p97 monomers within the ring allows the aromatic residues to function in a “staircase” or “conveyor belt” fashion, resulting in the effective translocation of oligo‐ubiquitinated substrates all the way through the pore of a Cdc48/p97/VCP hexamer to the opposite (trans/D2) side of the ring. Since the binding and translocation of the substrate is initiated by its ubiquitin moiety, the Cdc48/p97/VCP complex can process large and diverse groups of substrates, provided they are ubiquitinated as is the case for ERAD substrates (Ji *et al*, 2021). In parallel, Cdc48/p97/VCP may also serve as a “retrochaperone” for ubiquitinated membrane substrates, effectively promoting their solubility in the aqueous cytoplasm following retrotranslocation (Neal *et al*, 2017). This feature may be more important in yeast than in metazoans, as the latter also possess the multi‐subunit Bag6‐SGTA‐Trc35 complex, which similarly engages and promotes solubility of retrotranslocated substrates (Wang *et al*, 2011; Xu *et al*, 2012; Payapilly & High, 2014).

Along with substrates, the *cis*‐side of the Cdc48/p97/VCP complex also scaffolds other components such as yeast Otu1 and mammalian Yod1. These deubiquitinating enzymes have been shown to shorten the polyubiquitin chains on ERAD substrates, likely easing their translocation through the Cdc48/p97/VCP complex (Ernst *et al*, 2009; Stein *et al*, 2014), and may also prevent re‐binding of already translocated substrates (Ji *et al*, 2021). The association of other DUBs, such as Atx3, with Cdc48/p97/VCP suggests that ubiquitin chain trimming is a routine process for this platform (Wang *et al*, 2006). In mammalian cells, substrates emerging on the *trans*‐side of the Cdc48/p97/VCP complex with shortened ubiquitin chains are re‐ubiquitinated by association with the chaperone BAG6 and the ubiquitin ligase RNF126 (Hu *et al*, 2020). Other cytosolic ubiquitin ligases, such as UBE3C and UBR4, also appear able to act on ERAD substrates, and may serve a similar role (Leto *et al*, 2019; van de Weijer *et al*, 2020). They increase the efficiency of degradation of some ERAD substrates by adding branched and mixed ubiquitin chains, which facilitate fast access to the proteasome.

An outstanding conundrum is how N‐linked oligosaccharides, covalently modifying most putative ERAD substrates while in the ER lumen, could navigate through Cdc48/p97/VCP. Their extended structures would presumably hinder progress of a polypeptide through the narrow central pore. A solution to this apparent obstacle may come from recruitment of the cytosolic deamidating enzyme peptide *N*‐glycanase (NGly1) to Cdc48/p97/VCP by way of its PUB domain (Allen *et al*, 2006; Zhao *et al*, 2007). The NGly1 PUB domain binds to the D1 and/or D2 ATPase domains of p97/VCP, placing it at least in the vicinity of retrotranslocated (and glycosylated) substrates. If present, this enzyme could ensure that N‐linked sugars conjugated to most membrane and secreted proteins while in the ER are removed after retrotranslocation, but prior to engagement by Cdc48/p97/VCP. Consistent with this model, inhibition of the proteasome causes retrotranslocated ERAD substrates to accumulate in a deglycosylated form in the cytosol (Wiertz *et al*, 1996). However, removal of N‐linked glycans is not essential, as yeast cells lacking Png1 peptide *N*‐glycanase, or mammalian cells treated with the NGly1 inhibitor zVAD‐fmk, are still capable of degrading glycosylated ERAD substrates, albeit at lower efficiency (Suzuki *et al*, 2000; Grotzke *et al*, 2013). Whether glycans are in this case threaded through the pore, or slide in between the subunits of the Cdc48/p97/VCP hexamer as recently observed for certain ubiquitin molecules, is unclear (Ji *et al*, 2021).

### Parallel operations: how different ERAD branches divide the load

The ability for a wide and diverse range of substrates to be targeted by ERAD comes from its organization into multiple branches, each operating in parallel but with distinct specificity. Yet, the molecular determinants for dividing the labor among ERAD branches and the mechanisms of substrate preference and recognition of each branch remain, for the most part, elusive. It was first proposed that in yeast, with a simple ERAD system comprised of only three branches (Fig 1B), the differential selectivity for misfolded proteins is based on the compartment in which the lesion/misfolding occurs (Huyer *et al*, 2004; Vashist & Ng, 2004; Carvalho *et al*, 2006). This model posits that substrates exposing misfolded domains to the ER lumen (ERAD‐L substrates) or to the cytosol (ERAD‐C substrates) engage with ERAD branches using Hrd1 or Doa10, respectively. However, even in this simple eukaryote, the scenario becomes more complex when considering proteins with lesions in the membrane (ERAD‐M substrates), with all three yeast ERAD branches (Hrd1, Doa10, and Asi) able to target ERAD‐M substrates (Sato *et al*, 2009; Habeck *et al*, 2015; Ruggiano *et al*, 2016; Natarajan *et al*, 2020).

In mammals, more than 25 different E3 ubiquitin ligases reside in the ER, and at least a dozen of them have been implicated in ERAD (Fig 1B). Despite this expansion of available degradation routes, some aspects of substrate selectivity nevertheless remain evolutionarily conserved. For example, misfolded soluble substrates in the ER lumen (of the ERAD‐L variety) are exclusively processed by the HRD1 branch, as in yeast. However, the ERAD‐L/M/C classification, based on the location of the misfolded domain, appears an oversimplification when describing the complex and modular nature of ERAD organization in mammalian cells, which may leverage or prioritize other features to influence the engagement with particular ER‐E3s (Bernasconi *et al*, 2010). With membrane‐bound substrates, potentially accessible to every ER‐E3 through the phospholipid bilayer they share, those intrinsic features seem likely to be important in determining ER‐E3 selectivity as well as priority. So, while there will be instances of ER‐E3 exclusivity, as in the case of luminal substrates and Hrd1, other substrates are redundantly processed by multiple routes (see the example of HMGCR, below). While the large number of ER‐E3s in higher eukaryotes makes deconvolving redundancy challenging, new platforms that multiplex knockdowns/knockouts may help to shed light on functional overlap that may exist. Moreover, the rules and the molecular underpinnings governing the selectivity and preference of each ER‐E3 branch for different misfolded membrane proteins presented to them remain undefined. Some clarity into this complex scenario, however, comes from the regulated degradation of certain folded ER‐resident membrane proteins by ERAD. As detailed below for certain sterol metabolism enzymes, specific adaptors are involved in recognition and recruitment of substrates to a defined ERAD branch.

### Hrd1 ERAD branch: One way out (of the ER lumen)

ERAD regulates ER functions primarily via its role in the degradation of membrane proteins. However, the mechanism of ERAD is best understood for luminal (ERAD‐L) substrates, which are targeted to the Hrd1 complex in both yeast and mammals. The Hrd1 complex is conserved throughout eukaryotic evolution and constitutes the most extensively studied branch of ERAD. It plays a central role in maintaining ER homeostasis, with many of its key components under the transcriptional control of the unfolded protein response (UPR) (Friedlander *et al*, 2000; Fenech *et al*, 2020). Early studies in yeast revealed important aspects on how soluble substrates are recognized in the ER lumen and retrotranslocated across the membrane into the cytosol via a multi‐subunit, membrane‐spanning complex organized around the Hrd1 ubiquitin ligase (Carvalho *et al*, 2006; Denic *et al*, 2006; Gauss *et al*, 2006). These studies, primarily using the model substrate CPY*, a misfolded version of yeast carboxypeptidase (Hiller *et al*, 1996), were instrumental in defining the factors and mechanisms used by the Hrd1 complex to select/engage and retrotranslocate misfolded glycoprotein substrates across the ER membrane. As part of a multi‐component complex, Hrd1 is associated on either side of the ER membrane with additional proteins that provide functionalities essential for the ERAD process (Fig 2B). The Hrd1‐associated factors Hrd3 and Yos9 discriminate and engage misfolded glycoproteins in the ER lumen (Fig 2B). In mammals, this conserved process is carried out by the homologues Sel1L and OS‐9/XTP3‐B, respectively. Bipartite recognition of adjacent but distinct substrate features by these proteins marks substrate engagement in ERAD: While Hrd3/Sel1L, cooperating with Kar2/BiP, recognize and engage unstructured polypeptide regions, the lectin‐like binding domains of Yos9/OS‐9 and XTP3‐B selectively recognize N‐linked glycans with a terminal α1,6‐linked mannose residue (Quan *et al*, 2008; Hosokawa *et al*, 2009; Xie *et al*, 2009). In the “mannose timer” model, this glycan modification arises from prolonged exposure to a slow‐acting mannosidase enzyme in the ER lumen (Hebert & Molinari, 2012). It has also been proposed that substrate trimming may require traffic through an ill‐defined ER‐derived compartment containing this mannosidase activity (Benyair *et al*, 2015). Presence of this trimmed glycan on a nascent polypeptide is indicative of its long ER residence and of unsuccessful cycles of folding/refolding (Hebert & Molinari, 2012; Xu & Ng, 2015). Thus, by incorporating information on the folding state and residence time in the ER, the “degradation signal” recognized by the Hrd1 complex prioritizes terminally misfolded proteins while sparing both folded and newly synthesized molecules from degradation. This substrate recognition mechanism is conserved in mammalian cells, exemplified by studies on the degradation of NHK, the “null Hong Kong” mutant variant of human alpha‐1 antitrypsin (Christianson *et al*, 2008; Hosokawa *et al*, 2008). However, the presence of trimmed oligosaccharides is not an essential prerequisite, as misfolded, nonglycosylated substrates in the ER lumen are also targeted by the Hrd1 complex. How exactly such substrates are recognized is unclear, but Yos9 (OS‐9/XTP3‐B) does not appear to play a role (Leto *et al*, 2019). Instead, work in mammalian cells has shown that the ER luminal chaperones BiP and ERDJ5 as well as HERP, a subunit of the Hrd1 complex and likely the functional homologue of yeast Usa1, appear to be involved (Okuda‐Shimizu & Hendershot, 2007).

To reach the ubiquitination machinery, substrates recognized in the lumen must traverse the ER membrane into the cytosol. A path for substrate retrotranslocation across the lipid bilayer was recently revealed by improved cryo‐EM reconstructions of the yeast Hrd1 complex (Wu *et al*, 2020). These structures reveal that the membrane domains of Der1 and Hrd1 each harbor hydrophilic cavities facing the luminal and cytosolic sides of the membrane, respectively, thus forming two “half‐channels” (Fig 2B). Lateral gates in the Der1 and Hrd1 halves face each other in a thinned membrane region. Still, Der1 and Hrd1 are not directly docked to each other, suggesting that portions of the substrate may contact bilayer lipids during retrotranslocation. Together with earlier crosslinking experiments (Carvalho *et al*, 2010; Mehnert *et al*, 2014), these data support a model in which substrates recognized by Hrd3/Yos9 reach the Der1 luminal cavity in close proximity, subsequently inserting as a loop into the Hrd1/Der1 channel. The membrane thinning induced by Hrd1/Der1 could also be expected to facilitate retrotranslocation by lowering the energy barrier for moving hydrophilic amino acid residues through the hydrophobic interior of a lipid bilayer (Wu *et al*, 2020). As a portion of the substrate emerges on Hrd1 cytosolic cavity, its ubiquitination by the Hrd1 cytosolic RING domain (and E2s) would promote the engagement of Cdc48/p97/VCP for providing the ratcheting/pulling force necessary for extraction (discussed above). Subsequent rounds of ATPase hydrolysis by this complex then imposes directionality onto later stages of substrate retrotranslocation.

This current model suggests that the active channel is formed by a monomeric Hrd1 complex (Wu *et al*, 2020). However, biochemical studies on endogenous Hrd1 indicate that it is present in high molecular weight complexes corresponding to dimeric or even higher oligomeric states (Horn *et al*, 2009; Carvalho *et al*, 2010; Hwang *et al*, 2017). Whether these oligomers are involved in processing distinct substrates, or instead correspond to immature, idle, or regulatory forms of the Hrd1 complex, are important open questions. How Hrd1 channel gating is regulated also remains unresolved. Hrd1 channel activity in yeast was shown to be stimulated by self‐ubiquitination on one (or more) lysine residues within its RING domain as well as substrate engagement (Baldridge & Rapoport, 2016; Peterson *et al*, 2019; Vasic *et al*, 2020). But whether these events cause Hrd1 conformational changes or increase channel “activity” by some other mechanism is currently unknown. It remains to be confirmed whether auto‐ubiquitination is also essential for functionality of mammalian Hrd1, which notably has far fewer available lysine acceptors in its cytoplasmic domain, although one lysine within the catalytic RING domain is conserved throughout all metazoans. These efforts to decipher the molecular underpinnings of how Hrd1 complexes process lumenal substrates are slowly bringing the ERAD mechanism into greater focus. New structural techniques and reconstitution systems should help to accelerate this progress, allowing to fully visualize the dynamic nature of ERAD.

### Escape Options: Multiple exit routes from the ER membrane

Along with its exclusive role for ERAD‐L substrates, the Hrd1 branch of ERAD described above is also actively involved in the degradation of select ERAD‐M substrates. Notably however, there are several indications that the degradation path for membrane‐embedded substrates through Hrd1 is distinct from that taken by the soluble ERAD‐L substrates. First, yeast Hrd1 mutants compromised in their ability to process some ERAD‐M substrates remain competent to degrade those of ERAD‐L (Sato *et al*, 2009). Unlike ERAD‐L substrates, Hrd1—already co‐occupying the same membrane environment—does not necessarily require cofactors in order to engage ERAD‐M substrates, and may even play a direct role in their recognition (Sato *et al*, 2009). Second, retrotranslocation of membrane‐bound substrates appears to occur independently of Hrd1 self‐ubiquitination (Neal *et al*, 2018, 2020). Lastly, retrotranslocation of membrane‐bound substrates is independent of Der1 (Taxis *et al*, 2003; Vashist & Ng, 2004). Instead, Dfm1, a rhomboid‐pseudoprotease protein related to Der1, and able to replace Der1 in an alternative Hrd1 complex (Goder *et al*, 2008), has been implicated in the degradation of membrane proteins (Neal *et al*, 2018). Curiously, Dfm1 also facilitates Hrd1‐independent membrane protein retrotranslocation, by assisting other ERAD branches such as the Doa10 branch (Fig 2C) (Stolz *et al*, 2010; Avci *et al*, 2014; Neal *et al*, 2018). In fact, retrotranslocation of an engineered membrane substrate ubiquitinated independently of ERAD ubiquitin ligases appears to depend only on Dfm1 (Neal *et al*, 2018). A recent structure showed that Derlin1, the mammalian Dfm1 homologue, can assemble into tetramers suggesting that retrotranslocation of membrane proteins by these proteins may involve their oligomerization (Rao *et al*, 2021). The complex relationship between Hrd1, Der1, and Dfm1 in yeast, and the variations observed, raises intriguing questions of distinct functionalities associated with these factors. This is illustrated by the observation that yeast Dfm1 deletion strains can over time restore membrane protein degradation by duplicating chromosome XV, which contains Hrd1, or simply by overexpressing Hrd1 (Neal *et al*, 2018). How closely this represents the organization, regulation, and function within mammalian cells is an important question to address in the future, particularly in light of the increased number of participating factors.

The yeast ERAD branch defined by the Doa10 complex, composed of the ubiquitin ligase Doa10 and the ubiquitin conjugation enzymes Ubc6 and Ubc7, was initially identified through its role in degradation of the soluble transcriptional repressor Mat2α (Swanson *et al*, 2001). Doa10 and its mammalian equivalent TEB4/MARCHF6 also target other soluble proteins, which like Mat2α associate transiently with the cytoplasmic face of the ER via amphipathic helices (Ravid *et al*, 2006; Stefanovic‐Barrett *et al*, 2018). The Doa10 (or TEB4/MARCHF6) ERAD branch also engages membrane proteins, including various tail‐anchored (TA) proteins containing a single transmembrane segment near to the C‐terminus (Swanson *et al*, 2001; Habeck *et al*, 2015; Stefanovic‐Barrett *et al*, 2018; Dederer *et al*, 2019). Well‐studied examples include unassembled Sbh2, a subunit of the SEC translocon involved in protein import into the ER, and Ubc6, a subunit of the Doa10 complex, implying a level of autoregulation. Interestingly, ERAD degradation of Sbh2 and Ubc6 depends in both cases on their transmembrane region. However, while the tail‐anchor region is sufficient for Doa10‐mediated degradation of Sbh2, Ubc6 degradation also requires its own ubiquitin‐conjugation activity conferred by its cytosolic domain, suggesting that ubiquitin conjugation may occur intramolecularly in this case (Walter *et al*, 2001). Doa10 further promotes degradation of mislocalized peroxisomal TA proteins such as Pex15 (Dederer *et al*, 2019; Matsumoto *et al*, 2019). In this case, Pex15 mislocalized to mitochondria is removed from the outer membrane by the Msp1 ATPase and handed over to Doa10. Whether Doa10 recognizes mislocalized Pex15 while in the cytosol, upon its insertion into the ER membrane, or both is unclear. It is also unclear whether Doa10 recognizes Pex15 through its TA region. While these examples suggest a general role of Doa10 in quality control of TA proteins, our understanding of the molecular details involved in the recognition of these substrates remains incomplete.

Mammalian TEB4/MARCHF6 and a partly redundant ERAD ubiquitin ligase, TRC8/RNF139, are also implicated in quality control of TA proteins. Curiously, recognition by TEB4/MARCHF6 or TRC8 requires the transmembrane (TM) segment of these TA proteins to be cleaved by the intramembrane protease SPP (Signal Peptide Peptidase) (Boname *et al*, 2014; Chen *et al*, 2014; Stefanovic‐Barrett *et al*, 2018; Yücel *et al*, 2019). SPP‐mediated processing of TA substrates occurs within the plane of the membrane, likely on the lumen‐proximal leaflet, followed by ubiquitination via TEB4/MARCHF6 or TRC8. The large membrane domains of TEB4/MARCHF6 and TRC8, containing 14 and 12 predicted TM segments, respectively, might facilitate the retrotranslocation of the processed TA substrate with the aid of p97/VCP ATPase. It remains unclear how SPP initially selects its TA substrates and how the processed TA proteins are handed off to and subsequently recognized by TEB4/MARCHF6 and TRC8.

In addition to TA proteins, TEB4/MARCHF6 targets other membrane substrates. This includes degradation of the membrane domain of yeast Erg11 (Erg11TM), when expressed in mammalian cells (van de Weijer *et al*, 2020). In contrast to TA substrates, TEB4/MARCHF6‐dependent degradation of Erg11TM may not be redundant with TRC8 nor depend on prior substrate processing by SPP. However, as in the case of TA proteins, the TM segment of Erg11TM was found essential for degradation, suggesting a direct role of TEB4/MARCHF6 in TM domain recognition. TEB4/MARCHF6 is involved in degradation of unassembled FAM8A1, a membrane‐bound component of the Hrd1 complex, thus exemplifying co‐regulation between ER‐E3 complexes (Schulz *et al*, 2017). How Doa10 and TEB4/MARCHF6 recognize/engage substrates has yet to be established, but it appears to involve a conserved C‐terminal element (Zattas *et al*, 2016). The precise substrate feature(s) being recognized have not been defined either, yet it is apparent that not all TMs are being recognized. For example, CYP51A1TM, a model substrate analogous to Erg11TM and encompassing the TM domain of the human Erg11 homologue, is targeted to ERAD via the recently identified RNF185/MBRL complex and independently of TEB4/MARCHF6 (van de Weijer *et al*, 2020). At the core of this complex is the multi‐spanning membrane protein Membralin (MBRL or TMEM259), which independently binds to the ubiquitin ligase RNF185 and to either TMUB1 or TMUB2, members of the transmembrane and ubiquitin‐like domain‐containing protein family (van de Weijer *et al*, 2020). While more work is required to understand how RNF185/MBRL functions, recognition of CYP51A1TM was found to involve both its TM region as well as a short luminal amphipathic helix. This difference underscores how seemingly equivalent substrates have evolved to demand the broad range of metazoan E3s offered by evolutionary diversification.

## ERAD and regulation of ER functions

Investigations of ERAD go beyond the quest for components, their mechanistic organization, or roles in degradation of aberrant proteins. Seminal studies in yeast have shown that levels and stability of the key sterol biosynthesis enzyme, 3‐hydroxy‐3‐methylglutaryl‐CoA reductase (HMGCR), are controlled by ERAD, leading to the identification of Hrd1 (in fact, HRD stands for HMGCR Degradation) as its central component (Hampton *et al*, 1996). This early work demonstrated the role of ERAD in sterol homeostasis and its potential to regulate other aspect of ER physiology. Indeed, the search for other endogenous ERAD substrates, their regulation, and physiological relevance, is now an important and growing area of research. In this section, we discuss examples of key membrane proteins targeted by ERAD and how this mode of regulation affects cell physiology.

### Regulation of sterol biosynthesis by ERAD

**Figure 3:**
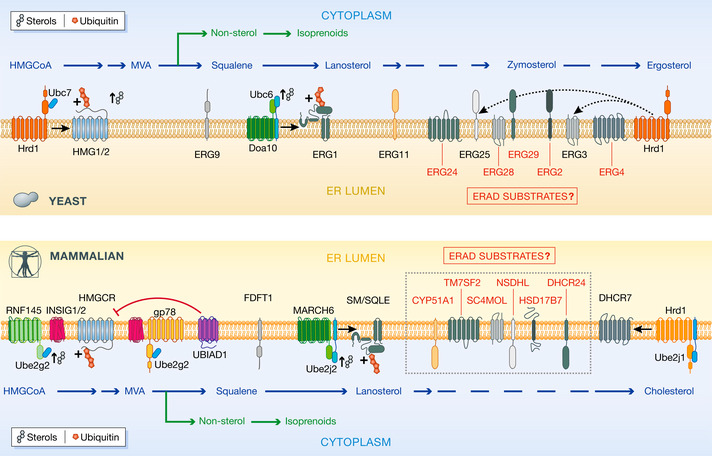
Regulation of sterol biosynthesis by ERAD Endoplasmic reticulum‐resident enzymes involved in sterol biosynthesis pathways of yeast (top) and mammals (bottom) are shown, along with the different ubiquitin ligases that regulate their abundance via ERAD. Sterol‐related biosynthetic products of each enzyme are shown (in blue) as are the nonsterol isoprenoids (in green). Enzymes whose regulation by ERAD has not yet been described are also shown (in red).

Sterol levels profoundly affect the properties of cellular membranes, and sterol biosynthesis through the mevalonate pathway demands precise regulation (Luo *et al*, 2020). In addition to cholesterol in mammals and ergosterol in yeast, the mevalonate pathway produces other essential metabolites such as vitamin K, ubiquinone, or steroid hormones. Coordinated synthesis of these various products depends on feedback regulation of rate‐limiting mevalonate pathway enzymes by ERAD‐dependent turnover (Fig 3). The best‐studied cases are mammalian HMGCR and SQLE (squalene monooxygenase), and their respective yeast counterparts Hmg2 and Erg1. In both yeast and mammalian cells, degradation of these enzymes involves two distinct ERAD branches. In yeast, high flux through the mevalonate pathway resulting in accumulation of intermediate metabolites such as farnesyl pyrophosphate, leads to Hmg2 downregulation by Hrd1‐dependent ERAD. High levels of geranylgeraniol pyrophosphate appear to destabilize the Hmg2 membrane domain, something that is perceived by the Hrd1 complex as a misfolded membrane protein and thus triggers its rapid degradation (Wangeline & Hampton, 2018). ERAD‐mediated regulation of mammalian HMGCR also occurs upon high flux through the mevalonate pathway, but the involvement of distinct signals and components reveals the evolution of more complex controls of this pathway in higher eukaryotes (Schumacher & DeBose‐Boyd, 2021). When sterol intermediates abound, HMGCR binds the integral ER membrane protein INSIG, whose association with several ERAD ubiquitin ligases facilitates ubiquitination and subsequent degradation of HMGCR (Sever *et al*, 2003a, 2003b). The precise identity of the ubiquitin ligases involved in HMGCR degradation has been a point of contention for some time, but recent genome‐wide CRISPR/Cas9 knockout screens indicate gp78 and RNF145 as the principal E3s, with only minor contribution by Hrd1 (Menzies *et al*, 2018). INSIG is itself subject to cholesterol‐regulated degradation via the gp78 ERAD branch, revealing an additional layer of complexity of HMGCR regulation (Lee *et al*, 2006). Furthermore, in the presence of high sterol levels, UBIAD1, a key enzyme in vitamin K biosynthesis, binds to the HMGCR/INSIG complex, attenuating degradation of ubiquitinated HMGCR (Schumacher *et al*, 2015). Membrane accumulation of the isoprenoid geranylgeranyl pyrophosphate triggers UBIAD1 release from this complex, restoring fast HMGCR degradation. Thus, ERAD‐mediated degradation of HMGCR is finely tuned by multiple mevalonate pathway metabolites and regulators.

Squalene monooxygenase constitutes another point in this pathway subjected to ERAD‐mediated feedback regulation. SQLE and Erg1 levels depend upon mammalian TEB4/MARCHF6 or its yeast homologue Doa10, respectively (Foresti *et al*, 2013; Zelcer *et al*, 2014). SQLE degradation is stimulated by high cholesterol concentrations and depends on an N‐terminal region in SQLE (Gill *et al*, 2011). Cholesterol‐induced membrane association of the SQLE N‐terminus in combination with increased stability of the ubiquitin ligase TEB4/MARCHF6 results in accelerated SQLE degradation (Chua *et al*, 2017; Sharpe *et al*, 2019). Degradation of yeast Erg1, which does not share the cholesterol‐responsive N‐terminus of SQLE, is instead stimulated by lanosterol (Foresti *et al*, 2013). However, the mechanisms sensing lanosterol concentration in the membrane have not yet been identified.

Other sterol‐biosynthetic enzymes found to be targeted for ERAD degradation include mammalian DHCR7 (Prabhu *et al*, 2016), DHCR14 (Capell‐Hattam *et al*, 2020), CYP51A1 (van de Weijer *et al*, 2020), and yeast Erg3 (Jaenicke *et al*, 2011), Erg11 (Foresti *et al*, 2014) and Erg25 (Buck *et al*, 2020; Christiano *et al*, 2020). However, the impact of these proteolytic events on sterol homeostasis, or whether additional enzymes in the pathway are also targets for ERAD, remains to be fully ascertained. Regardless, ERAD appears central to the complex regulation of sterol homeostasis necessary for viability in all eukaryotes, which suggests that co‐evolution may have occurred to enable the dynamic regulation of this essential biosynthetic pathway.

### Border patrol: the maintenance of ER membrane identity

ERAD contributes to ER membrane identity by promoting the degradation of proteins mislocalized to the ER. Among this class of ERAD substrates are proteins normally localized to lipid droplets (LDs) (Olzmann & Carvalho, 2019). These storage organelles essential for lipid homeostasis consist of a hydrophobic core filled by neutral lipids, enclosed by a unique phospholipid lipid monolayer that often remains continuous with the outer leaflet of the ER membrane. The specialized membrane bridges allow the trafficking of molecules between the two organelles, including some hairpin‐containing membrane proteins. By degrading excess amounts of these proteins in the ER, ERAD fine‐tunes the overall levels of certain LD proteins both in yeast (Ruggiano *et al*, 2016) and mammalian (Bersuker *et al*, 2018) cells.

**Figure 4:**
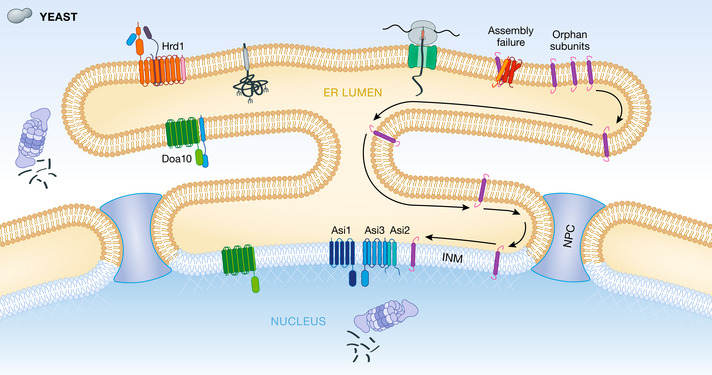
Asi‐mediated transmembrane domain recognition at the inner nuclear membrane Selected TM domain‐containing proteins failing to assemble appropriately into oligomeric complexes in yeast can diffuse through the ER membrane (in brown) and past the nuclear pore complex (NPC) to the inner nuclear membrane (in blue). There, the Asi complex (Asi1/2/3) engages these orphan subunits through Asi2‐mediated recognition of the substrate's TM domain, leading to subsequent ubiquitination and degradation via nuclear‐localized proteasomes.

Another example is the inner nuclear membrane (INM), a specialized domain of the ER that together with the outer nuclear membrane forms the nuclear envelope. While contiguous with the remaining ER membrane, the INM houses a specialized set of proteins involved in nuclear organization and functions (Pawar & Kutay, 2021). Work in yeast has revealed the Asi complex as an ERAD branch restricted to the INM (Foresti *et al*, 2014; Khmelinskii *et al*, 2014). The Asi complex is composed of three integral membrane proteins: two ubiquitin ligases, Asi1 and Asi3, and Asi2, a recognition factor for select substrates, which act together to preserve the unique INM proteome by degrading mislocalized ER membrane proteins (Fig 4). Consistent with this function, no *bona fide* INM resident protein has been found to be targeted by the Asi complex. Among mislocalized Asi substrates, the best characterized are unassembled subunits of established ER membrane protein complexes, such as subunits of oligosaccharyltransferease (OST) essential for N‐linked protein glycosylation (Natarajan *et al*, 2020). How mislocalized proteins are specifically recognized, and the mechanisms of Asi‐mediated retrotranslocation, are important open questions. Asi complex homologues are present only in some fungi, and it is not yet clear if an equivalent INM‐specific ERAD branch exist in higher eukaryotes. Certain ubiquitinated INM proteins in mammalian cells, such as disease‐associated mutant forms of the lamin‐B receptor, are indeed extracted from the INM by p97/VCP for degradation by the proteasome (Tsai *et al*, 2016). However, the components involved in the recognition and ubiquitination of these mammalian INM substrates have not been identified. While the role of ERAD in controlling the lateral organization of ER domains is still emerging, these studies suggest that besides folding states, other protein properties, such as their spatial distribution, can be monitored by ERAD to enforce organelle integrity.

### Regulation of inter‐organelle interfaces

**Figure 5:**
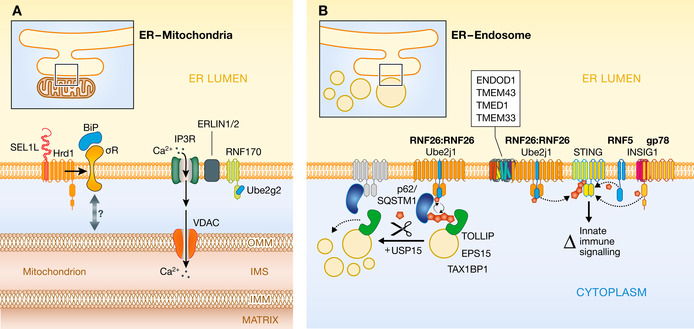
Regulation of organelle interfaces by ER‐resident E3s (A) Two examples of ER‐resident ubiquitin ligase complexes regulating proteins at the ER‐mitochondria interface. Hrd1‐SEL1L impact abundance of the Sigma receptor (left) while RNF170, bridged by Erlin1/2, ubiquitinates IP3 receptors that regulate calcium ion efflux from the ER to the mitochondria through VDAC (right). (B) Regulation of endosomal positioning relative to the ER through ubiquitination of p62/SQSTM by the ER‐resident E3 RNF26 and E2 UBE2J1 (left). Ubiquitinated p62/SQSTM binds to endosomal proteins (e.g., TOLLIP, EPS15, TAX1BP1) to form a perinuclear endosomal cloud, which can be dispersed by the activity of the USP15 deubiquitinase. RNF26 is also, along with RNF5 and gp78/INSIG1, involved in ubiquitination of the innate immune signaling molecule STING when present in the ER membrane. Together with several interacting cofactors (TMEM43, TMEM33, TMED1, ENDOD1), RNF26 modulates the magnitude of interferon signaling mediated through the cGAS‐STING pathway.

Cellular organelles communicate at membrane contact sites (MCS)—regions where the membranes of distinct organelles come into close apposition (Wu *et al*, 2018; Scorrano *et al*, 2019). The ER establishes MCS with virtually all other cellular organelles and the plasma membrane (Valm *et al*, 2017; Shai *et al*, 2018). These MCS are often driven by membrane proteins, are highly dynamic in response to cellular environment, and enable the exchange of various signals or molecules, such as phospholipids, sterols, or calcium. Recent work has shown that tissue‐specific deletion of HRD1 or its partner SEL1L in mouse brown adipocytes leads to enlarged mitochondria whose MCS with the ER are greatly expanded (Zhou *et al*, 2020). These aberrant ER‐mitochondria MCS resulted in mitochondrial defects and an inability to maintain body temperature during cold conditions in this mouse model. Defective degradation of the ER membrane protein SigmaR1 in the ERAD‐deficient cells may in part explain this phenotype (Fig 5A) (Zhou *et al*, 2020). How SigmaR1 degradation controls the ER‐mitochondria contacts, and how the Hrd1 complex recognizes this substrate during cold adaptation, are outstanding questions to be answered with future studies.

Endoplasmic reticulum‐mitochondria contacts are also facilitated by interaction between inositol‐1,4,5‐triphosphate (IP3) receptors (IP3Rs) in the ER membrane and the voltage‐dependent anion channels (VDAC) in the mitochondria outer membrane (Szabadkai *et al*, 2006). IP3Rs are tetrameric ion channels that release calcium ions (Ca^2+^) from the ER lumen when activated, often triggered by IP3 released via activated cell surface receptors. Calcium released by activated IP3Rs into the cytoplasm serves various purposes, including triggering signaling cascades in mitochondria via interactions with VDAC. Like many sterol‐related enzymes, IP3R abundance is regulated by ERAD (Fig 5A). In particular, activated IP3Rs are targeted for degradation by the E3 ligase RNF170, with interactions mediated by Erlin1/2 (Pearce *et al*, 2009; Lu *et al*, 2011). Conformational changes involved in IP3R activation allow for its recognition by the RNF170/Erlin1/2 complex, resulting in rapid IP3R ubiquitination and proteasomal degradation, consequently decreasing ER calcium release. This adaptive mechanism in response to persistent activation of IP3‐dependent signaling pathways appears particularly important in certain tissues, such as in the central nervous system. Consistently, mutations in IP3R, Erlins, and RNF170 are associated with neurological disorders such as spastic paraplegias (Gao & Wojcikiewicz, 2020). Thus, the degradation of IP3Rs may offer another example of how ERAD impacts on organelle contact sites.

Conspicuous MCS between the ER and endosomes are important for lipid homeostasis, cargo sorting within endosomes, as well as for endosome fission, dynamics, and positioning (Wu *et al*, 2018). The ER‐resident ubiquitin ligase RNF26 appears to play a particularly prominent role in endolysosome perinuclear positioning (Fig 5B) (Jongsma *et al*, 2016). RNF26 is a polytopic membrane protein with a cytosolic RING domain at its very C‐terminus. It forms a dimer, is highly auto‐ubiquitinated, and assembles with a collection of other ER cofactors (Fenech *et al*, 2020). RNF26 works together with UBE2J1, the tail‐anchored, ER‐resident E2 principally associated with the Hrd1 branch of ERAD (Cremer *et al*, 2021). RNF26 and UBE2J1 cooperate to conjugate ubiquitin to the scaffold protein p62/SQSTM, facilitating engagement of the latter factor by ubiquitin‐binding proteins present in endosomal membranes (such as EPS15, TOLLIP, TAX1BP1) (Jongsma *et al*, 2016; Cremer *et al*, 2021). This allows endosomes to accumulate in a perinuclear region and limits the deployment of secretory cargo, such as growth factor receptors/EGFR (Cremer *et al*, 2021). The impact of RNF26 ubiquitination counteracted by the deubiquitinating activity of USP15 (Jongsma *et al*, 2016). While the process of ERAD‐mediated p62/SQSTM ubiquitination thus does not involve proteasomal degradation, it nevertheless highlights how RNF26 and UBE2J1 at the ER membrane exert spatiotemporal control over ER‐endosome contacts, influencing organelle architecture, transport dynamics, and function.

In an ERAD‐like functionality, RNF26 has also been implicated in controlling abundance of the innate immune signaling molecule STING/TMEM173 (Qin *et al*, 2014; Fenech *et al*, 2020). It is unclear whether this role for RNF26 is entirely separate to that of endosomal positioning, but the dual role being played via either degradative or nondegradative ubiquitination raise interesting questions about a multifunctional nature of some ER‐resident E3s. In fact, the ER‐E3s RNF5 and gp78 (via INSIG1) were also reported to ubiquitinate STING/TMEM173 when present in the ER, impacting its ability to initiate downstream signaling (Qin *et al*, 2014; Wang *et al*, 2014). Deconvolving the competing (or complementary) activities of ER‐resident E3s toward STING, and their impact on innate immune signaling, will be important to fully appreciate how ubiquitination dynamically governs activation of this key signaling pathway.

### Outlook

Genetic and biochemical studies over the past three decades have led to the identification of many ERAD components, and to an understanding of how they organize to productively target substrates for degradation. A current challenge is to dissect the molecular functions of many of these components, and their mechanisms of substrate processing. The rules guiding substrate selection also remain elusive for most ERAD substrates. Recent advances in cryo‐EM‐based structural analyses hold the promise to solve some of these issues, as shown by the discussed landmark structure of the yeast Hrd1 complex. Elegant efforts for reconstituting ERAD processing *in vitro* are also providing valuable mechanistic insight. Continuing to refine the structures and reconstituting other ERAD complexes with their full complement of endogenous components will clearly be a major step forward for this field. Moreover, including substrates in these structural reconstructions will add clarity to our understanding of the dynamic processivity underlying the ERAD mechanism.

Early indications of regulatory functionality for ERAD have been recently confirmed and expanded. It is now thought that ERAD machineries are extremely versatile and have evolved to monitor the levels of specific proteins or even distinct pools of a given protein, enabling control over multiple functions of the ER and how it relates to other cellular organelles. These regulatory ERAD functions are often context‐specific, and likely relevant in specific but not all cell types (reviewed in (Bhattacharya & Qi, 2019). Thus, this emerging area of ERAD investigation will benefit from studies using tissue‐specific animal genetic models or specialized cell systems. As the mechanistic and physiological insights into ERAD begin to coalesce, it bodes well for this vibrant field to continue to excel, with much more excitement still to come.
